# Increased predominance of HIV-1 CRF01_AE and its recombinants in the Philippines

**DOI:** 10.1099/jgv.0.001198

**Published:** 2019-01-24

**Authors:** Yue Chen, Bhavna Hora, Todd DeMarco, Regina Berba, Heidi Register, Sylvia Hood, Meredith Carter, Mars Stone, Andrea Pappas, Ana M. Sanchez, Michael Busch, Thomas N. Denny, Feng Gao

**Affiliations:** ^1^​ Department of Medicine, Duke Human Vaccine Institute, Duke University Medical Center, Durham, NC 27710, USA; ^2^​ I-REACT Clinic, Section of Infectious Diseases, Department of Medicine, The Medical City, 1605 Pasig City, Philippines; ^3^​ Blood Systems Research Institute, San Francisco, CA 94118, USA

**Keywords:** HIV-1, NFLG, subtype, tMRCA, DRMs, Philippines

## Abstract

The growth rate of new HIV infections in the Philippines was the fastest of any countries in the Asia-Pacific region between 2010 and 2016. To date, HIV-1 subtyping results in the Philippines have been determined by characterizing only partial viral genome sequences. It is not known whether recombination occurs in the majority of unsequenced genome regions. Near-full-length genome (NFLG) sequences were obtained by amplifying two overlapping half genomes from plasma samples collected between 2015 and 2017 from 23 newly diagnosed infected individuals in the Philippines. Phylogenetic analysis showed that the newly characterized sequences were CRF01_AE (14), subtype B (3), CRF01/B recombinants (5) and a CRF01/CRF07/B recombinant (1). All 14 CRF01_AE formed a tight cluster, suggesting that they were derived from a single introduction. The time to the most recent common ancestor (tMRCA) for CRF01_AE in the Philippines was 1995 (1992–1998), about 10–15 years later than that of CRF01_AE in China and Thailand. All five CRF01/B recombinants showed distinct recombination patterns, suggesting ongoing recombination between the two predominant circulating viruses. The identification of partial CRF07_BC sequences in one CRF01/CRF07/B recombinant, not reported previously in the Philippines, indicated that CRF07_BC may have been recently introduced into that country from China, where CRF07_BC is prevalent. Our results show that the major epidemic strains may have shifted to an increased predominance of CRF01_AE and its recombinants, and that other genotypes such as CRF07_BC may have been introduced into the Philippines.

## Introduction

Global efforts to strengthen HIV prevention and treatment programmes have reduced the transmission of HIV. However, whereas the growth in the number of HIV infections is decreased in many countries, Philippines has the fastest growth rate in the Asia-Pacific region, up by 200 % from 2010 to 2016 [1]. One of the main reasons for the sharp increase in the number of HIV infections in the Philippines is most likely the inadequate education and health promotion policy provided to the population, especially to the key at-risk populations: men who have sex with men (MSM), transgender women who have sex with men (TGW) and injection drug use (IDU) [2]. In 2016, 83 % of new HIV-1-infection cases were among MSM and TGW, most of whom were aged between 15 and 24 years [2]. The Philippines is facing a huge challenge to the fight against HIV [2, 3].

Since the first patient with AIDS in the Philippines was reported in 1984 [4], several HVI-1 subtypes (B, C, D and G), circulating recombinant forms (CRFs: CRF01_AE and CRF02_AG) and unique recombinants (01B and others) have been reported in that country [4–8]. These early studies showed that subtype B was the most prevalent HIV-1 strain (70 %) followed by CRF01_AE (20 %), while others accounted for smaller percentages. However, one recent study of *pol* gene sequences showed that CRF01_AE has become predominant (77 %) while the proportion of subtype B has decreased (22 %) [9]. All previous molecular epidemic surveys were carried out based on analysis of partial *gag*, *pol* or *env* sequences. Thus, the distribution of subtypes or CRFs in the Philippines may not be accurately accounted for, since the larger portion of the viral genome was not analysed. Thus, it is important to characterize HIV-1 whole-genome sequences to better understand whether, in the Philippines, new recombinants have been generated and become prevalent strains.

To better understand what viruses are circulating in the Philippines, we analysed near-full-length genome (NFLG) sequences from 23 HIV-1-infected individuals. Genetic analyses showed that CRF01_AE was predominant (61 %) and unique recombinants accounted for 26 %, while subtype B comprised only 13 % of the virus population involved. Our results indicate that CRF01_AE has become predominant, and its recombination with other circulating strains are increasing in frequency in the Philippines.

## Methods

### Participants

Patients newly diagnosed with HIV-1 infection in Medical City, which is an 800-bed hospital with an established Department of Health-accredited HIV treatment clinic, located in the National Capital Region of the Philippines, were invited to participate in this study during their first clinic visit in the period 2015–2017. All study participants, except one, were single Filipino males ages 22–42 years (mean age 29.21 years ±SD 5.33), from the following provinces: Bulacan (2), Capiz (1), Cavite (1), Cebu (1), Laguna (2) and Rizal (1); and from the following cities: Makati (2), Malabon (1), Mandaluyong (2), Manila (2), Pasig (3) and Quezon City (5). All patients were treatment-naive at the time of recruitment. Eighteen (78 %) reported homosexual transmission. All patients denied use of intravenous drugs. The mean CD4 count was 294.17±180.37 ml^–1^, with seven (30 %) having a CD4 count <200 ml^–1^. Plasma samples were collected from 23 subjects. Written informed consent was obtained from all participants. The study was approved by The Medical City Institutional Review Board and by the Duke University Institutional Review Board.

### Amplification of near-full-length HIV-1 genome

Viral RNA was extracted from 400 µl of each plasma sample using EZ1 Virus Mini Kit v2.0 (Qiagen, Valencia, CA) and used for cDNA synthesis using Superscript III Reverse Transcriptase (Invitrogen, Carlsbad, CA) with primers 1 .R3.B3R (5'-ACTACTTGAAGCACTCAAGGCAAGCTTTATTG−3' HXB2 nt9611-9642) and 07Rev9 (5′-CTTCCTGCCATAGGAGATGCCTAA-3' nt 5957–5980) for 3'- and 5'-half HIV-1 genomes, respectively. The 3'-half and 5'-half genomes of each virus were obtained by bulk PCR amplification as previously described [10]. All Near-full-length genome (NFLG) sequences one (1008) were obtained from plasma samples. The NFGL sequences of 1008 were amplified from a culture supernatant obtained after short-term culture of the plasma sample with peripheral blood mononuclear cells (PBMC) from HIV-1-negative donors as previously described [10].

### Sequence analysis

PCR amplicons were quantified using qPCR with the KAPA Library Quantification Kit Illumina platform (Kapa Biosystems, Wilmington, MA). The PCR amplicon from each sample was barcoded and then sequenced on MiSeq (Illumina, San Diego, CA) using the MiSeq Reagent Nano kit v2 (300 bp). The average coverage per base was 500–8000. The final consensus sequence from each library was obtained by assembling raw sequence reads using either Geneious software (Biomatters, Auckland, New Zealand) or High-performance Integrated Virtual Environment (HIVE) [11].

The final sequences were aligned together with subtype reference sequences from the Los Alamos HIV Sequence Database (www.hiv.lanl.gov) using clustal W [12], and manual adjustment for optimal alignment was done using SEAVIEW. Subtypes of newly characterized HIV-1 genomes were determined by phylogenetic tree analysis using the neighbour-joining (NJ) method with the Kimura two-parameter model [13, 14], and the reliability of topologies was estimated by bootstrap analysis with 1000 replicates. Recombination patterns in newly characterized HIV-1 genomes were initially analysed by the jumping profile Hidden Markov Model (jpHMM; http://jphmm.gobics.de/submission_hiv.html) [15]. The recombination breakpoints were confirmed by BootScan implemented in Simplot version 3.5.1 [16]. The recombination pattern of each virus was illustrated using RecDraw [17].

### Molecular evolution clock analysis

The divergence times for CRF01_AE were estimated using the Bayesian Markov chain Monte Carlo (MCMC) approach available in the package BEAST v1.8.2. The relaxed (uncorrelated log-normal) molecular clocks were enforced under the HKY nucleotide substitution models [18], with a gamma-distribution model of among-site rate heterogeneity (with four rate categories) [19]. Each MCMC analysis was run for 50 million steps and sampled every 10 000 states. Posterior probabilities were calculated with a 10 % burn-in and checked for convergence using Tracer v1.6. The maximum clade credibility tree was generated using Tree Annotator v1.8.2, available in BEAST, and FigTree 1.4.2 was used for visualization of the annotated trees [20].

### Genotypic analysis of drug resistance mutations

The raw sequence reads generated from MiSeq (Illumina, San Diego, CA) were uploaded to the HyDRA website [21]. All HIV drug resistance (HIVDR) mutations found in the *pol* genes – *protease* (PR), *reverse transcriptase* (RT) and *integrase* (IN) – are reported according to classifications outlined in the Stanford HIV Drug Resistance Database (https://hivdb.stanford.edu/) [22].

### Nucleotide sequence accession numbers

The GenBank accession numbers for the newly characterized sequences are MH327744-MH327766.

## Results

### Determination of infection stages

Fiebig stages of HIV-1 infection were determined based on the detection of viral genomes and HIV-1-specific antibodies in plasma as previously described [23]. Three samples were collected at Fiebig stage IV, two at Fiebig stage V, and 18 at Fiebig VI (Table 1). Recent (≤130 days) and long-term (>130 days) infection stages of these samples were also determined by limiting-antigen avidity (LAg) assay [24]. Seventeen were long-term infections (LT) while five were recent infections (Table 1). There was insufficient plasma from participant 1011 for the LAg assay. The recent infection stages as determined by Fiebig staging and LAg methods were in agreement in general. Among five recent infection cases as determined by LAg assay, one was at Fiebig stage IV (~31 days post infection) and the other four were at Fiebig stage VI (open-ended). All other Fiebig stage VI samples were classified as long-term infection by LAg assay. However, three out of four Fiebig stage IV and V (~100 days post infection) samples were classified as long-term infection by LAg assay.

**Table 1.** Demographic characteristics of HIV-1-infected individuals in the Philippines

Subject	Subtype	City	Gender	Age	Marital status	Collection date	Viral load (copies ml^–1^)	CD4 Count (cells per mm^3^)	Fiebig stage	LAg classification	Transmission route
1001	URF_01B	Makati	Male	23	Single	7/18/15	277 000	410	VI	LT	MSM
1002	B	Makati	Male	30	Single	7/20/15	161 000	282	VI	LT	MSM
1003	B	Bulacan	Male	36	Single	8/25/15	730 000	222	VI	Recent	na
1005	URF_01B	Quezon	Male	29	Single	10/7/15	133 000	514	VI	LT	MSM
1006	CRF01_AE	Laguna	Male	32	Single	10/26/15	303 500	88	VI	LT	MSM
1007	CRF01_AE	Laguna	Male	31	Single	10/26/15	50 000	450	VI	LT	MSM
1008*	CRF01_AE	Pasig	Male	23	Single	12/7/15	197 000	510	VI	Recent	na
1009	URF_01B	Cebu	Male	35	Single	2/9/16	570 000	240	VI	Recent	MSM
1010	CRF01_AE	Malabon	Male	21	Single	2/10/16	360 000	523	IV	Recent	MSM
1011	URF_01B	Bulacan	Male	27	Single	9/30/15	81 500	122	V	na	MSM
1012	CRF01_AE	Quezon	Male	34	Single	3/14/16	560 000	579	IV	LT	MSM
1013	CRF01_AE	Manila	Male	30	Single	3/15/16	300 000	34	VI	LT	MSM
1021	CRF01_AE	Pasig	Male	30	Married	1/16/17	174 000	64	V	LT	MSM
1022	B	Quezon	Male	30	Single	12/5/16	142 500	120	VI	LT	MSM
1023	URF_0107B	Quezon	Male	28	Single	2/20/17	205 000	249	VI	LT	MSM
1024	CRF01_AE	Rizal	Male	22	Single	9/21/16	69 500	285	VI	LT	Heterosexual
1025	CRF01_AE	Cavite	Male	33	Single	12/5/16	102 500	348	VI	Recent	Heterosexual
1026	CRF01_AE	Capiz	Male	24	Single	3/22/17	295 000	10	VI	LT	MSM
1027	CRF01_AE	Mandaluyong	Male	36	Single	10/5/16	318 000	182	IV	LT	MSM
1028	CRF01_AE	Quezon	Male	24	Single	2/8/17	165 000	420	VI	LT	MSM
1029	CRF01_AE	Pasig	Male	28	Single	11/9/16	69 500	512	VI	LT	MSM
1030	URF_01B	Mandaluyong	Male	42	Single	8/24/16	118 500	133	VI	LT	MSM
1031	CRF01_AE	Manila	Male	24	Single	9/7/16	237 000	469	VI	LT	Bisexual

* PBMC-derived viruses; LT, long-term infection; Recent, recent infection; MSM, men who have sex with men; na, data not available.

### Predominant CRF01_AE sequences are monophyletic in the Philippines

**Fig. 1.**
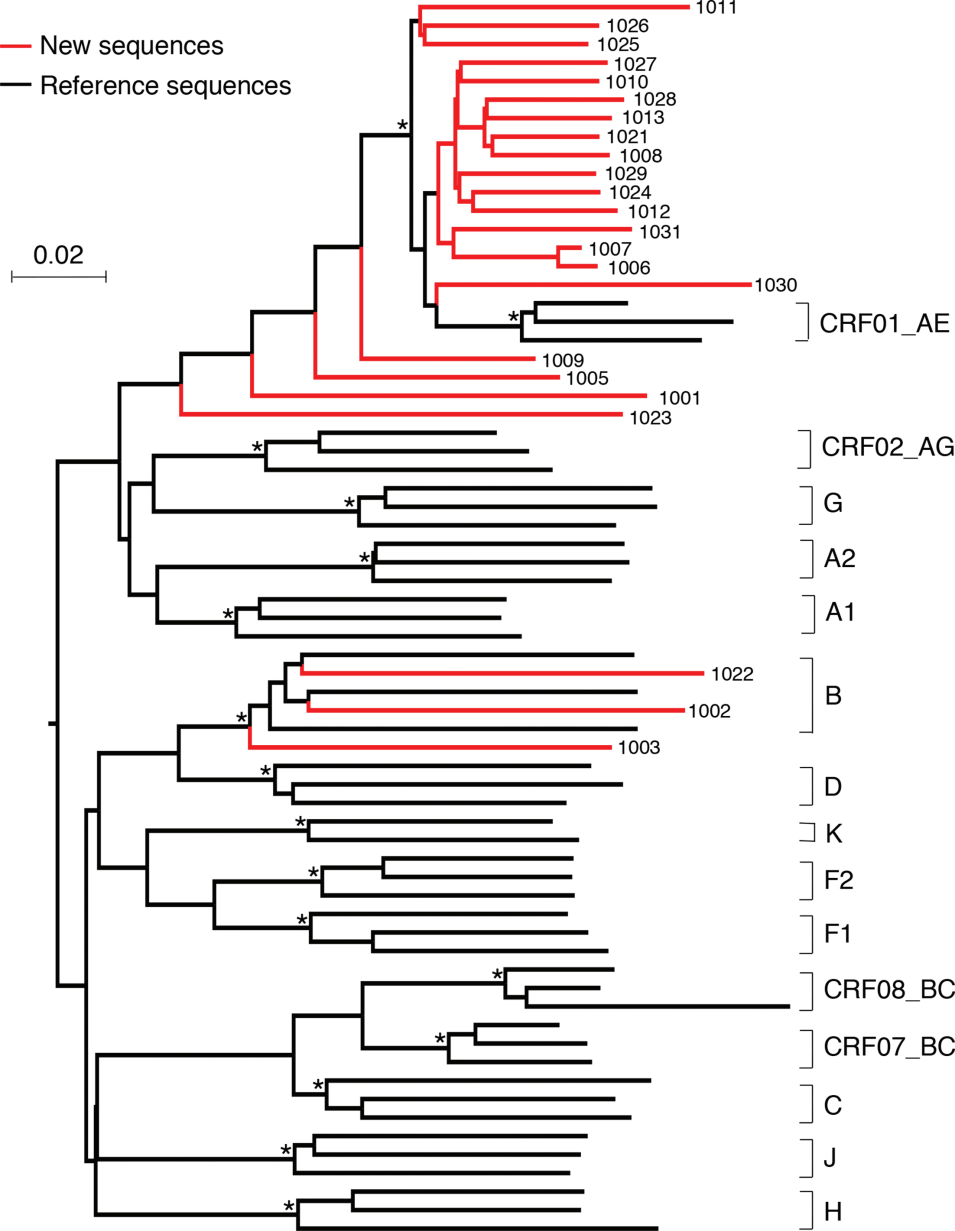
Phylogenetic analysis of near-full-length genome sequences. Newly obtained NFLG sequences from 23 HIV-1-infected individuals in the Philippines were aligned together with reference sequences from the HIV-1 sequence database (www.hiv.lanl.gov). The phylogenetic tree was constructed using the neighbour-joining method and Kimura two-parameter model. The scale bar represents 0.02 nucleotide substitutions per site. Asterisks indicate bootstrap values in which the cluster to the right is supported in 80 % or more replicates (out of 1000). The newly characterized viral sequences are shown in red, and other subtype reference sequences in black.

The NFLG sequences were obtained from 22 plasma samples by amplifying two overlapping half genomes. For the remaining sample (1008), which was negative for PCR amplification, the virus was isolated by PBMC co-culture from plasma. The NFLG sequence was obtained from viruses in cell culture supernatants by PCR amplification of two overlapping half genomes. The initial phylogenetic analysis of 23 near-full-length genome sequences showed that 20 newly characterized sequences clustered to the CRF01_AE references sequences, while three other sequences clustered closely to subtype B references sequences (Fig. 1).

**Fig. 2.**
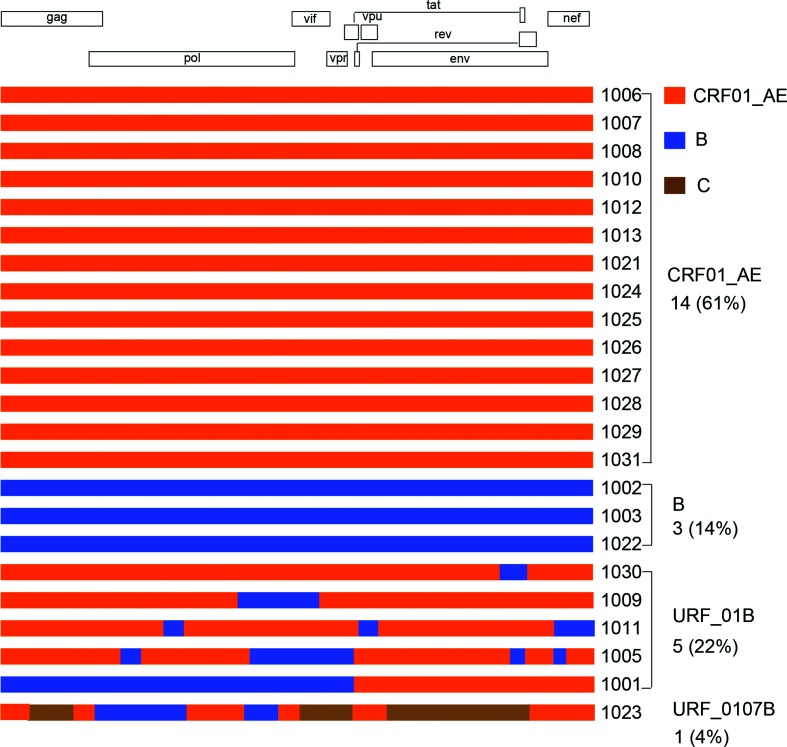
Recombination breakpoint analysis of NFLG sequences. Recombination breakpoints of the newly obtained NFLG sequences were determined using similarity plot and jpHMM. The recombination pattern for each NFLG sequence is shown using RecDraw. Sequences CRF01_AE, subtype B and CRF07_BC are indicated by orange, blue and brown boxes, respectively.

Some of the sequences either clustered far outside of the CRF01_AE clade or had longer branches than others (Fig. 1). To investigate whether such sequences were the result of recombination among different subtypes, we performed recombination analysis of these sequences using the tools jpHMM and BootScan. This further analysis showed that these sequences were indeed recombinants: five (22 %) CRF01/B recombinants and one (4 %) CRF01/CRF07/B recombinant, while 14 (61 %) were CRF01_AE and three (13 %) were subtype B (Fig. 2).

**Fig. 3.**
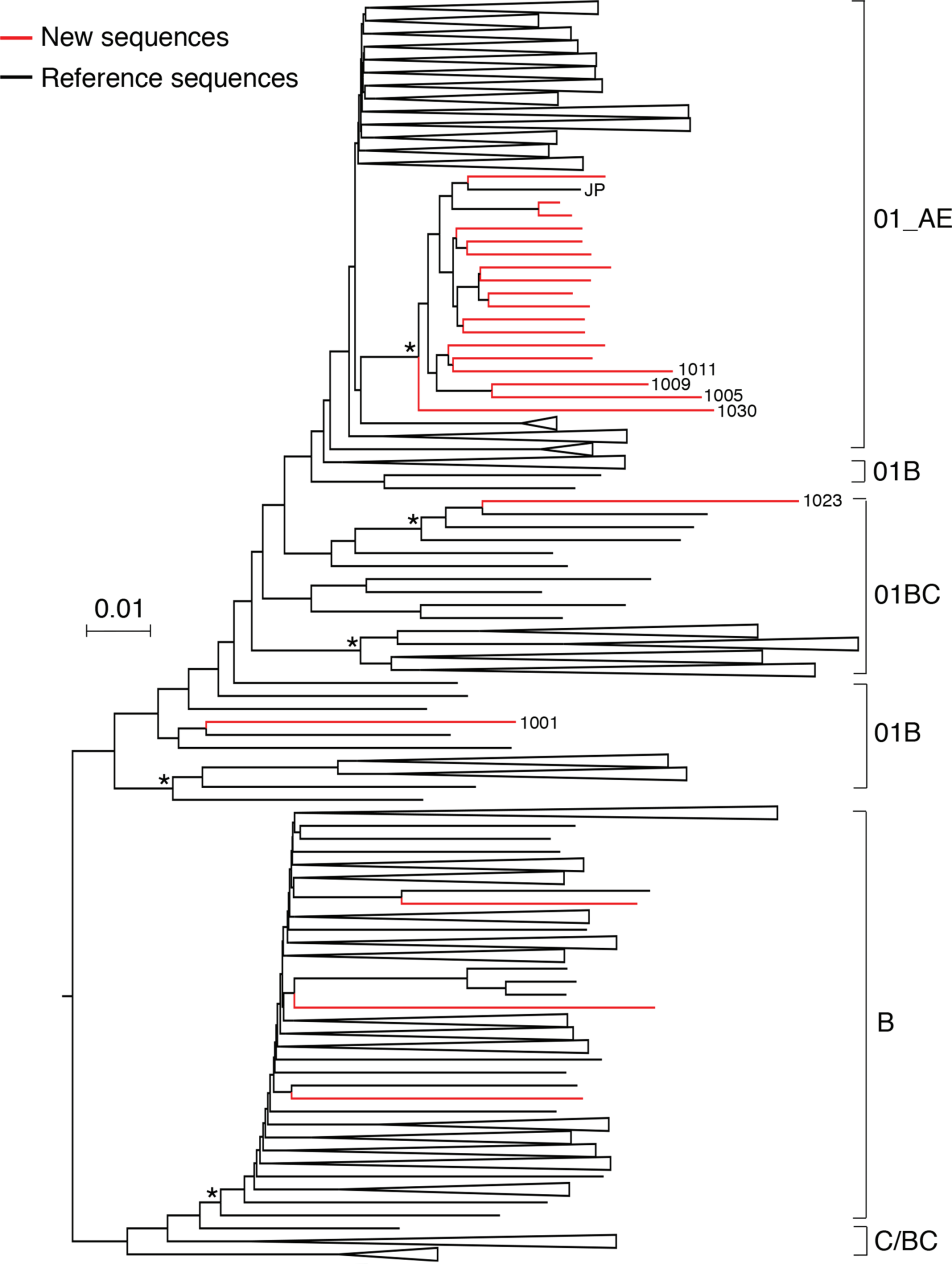
Phylogenetic analysis of new NFLG sequences with additional reference sequences. The 23 new NFLG sequences from the Philippines were aligned with additional CRF01_AE, CRF07_BC, CRF08_BC, subtype B and other reference sequences. The phylogenetic tree was constructed using the neighbour-joining method and the Kimura two-parameter model. The scale bar represents 0.02 nucleotide substitutions per site. Asterisks indicate bootstrap values in which the cluster to the right is supported in >80 % of replicates (per 1000). Newly characterized sequences in the Philippines are shown in red. The recombinant sequences are indicated by sample ID, and the sequence derived from Japan is indicated by JP. Sequences within a smaller cluster were collapsed and are shown as a triangle.

Interestingly, all 14 CRF01_AE sequences formed a tight cluster, indicating that these might have been derived from the same common CRF01_AE ancestor (Fig. 1). Compared to new CRF01_AE sequences, new subtype B sequences were more divergent and intermingled with subtype B reference sequences, suggesting that subtype B viruses in the Philippines were probably derived from multiple ancestors (Fig. 1). To further confirm our observation, we constructed a phylogenetic tree with our newly characterized sequences and hundreds of available NFLG CRF01_AE, subtype B, CRF01/B and CRF01/B/C sequences from the HIV sequences database. All new CRF01_AE sequences, together with four (1005, 1009, 1011 and 1030) CRF01/B recombinants, which contained only small subtype B portions, formed a tight cluster (Fig. 3). One CRF01_AE sequence from Japan clustered with all the newly characterized sequences. In contrast, all three new subtype B sequences remained intermingled with subtype B reference sequences.

**Fig. 4.**
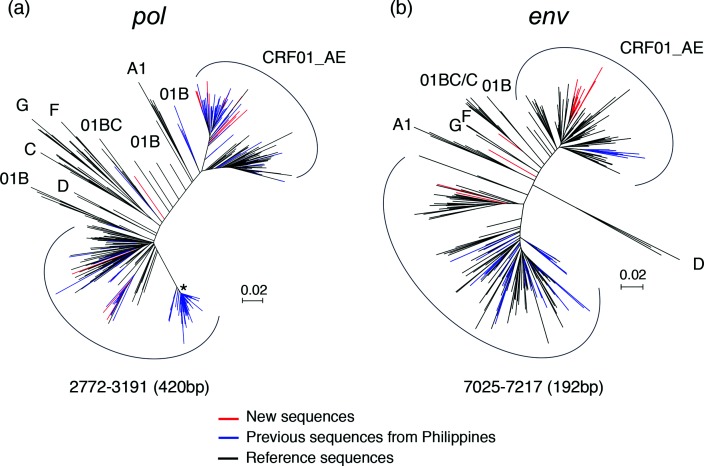
Phylogenetic analysis of partial *pol* and *env* gene sequences. Partial *pol* (left) and *env* (right) sequences available from the Philippines in the HIV sequence database were analysed, together with the newly characterized NFLG sequences and reference sequences. Since the sequences in each region were from different studies and did not fully overlap, neighbour-joining trees were constructed to include as many sequences as possible and to maximally utilize the sequence length for all available sequences. The CRF01_AE sequences from this study and from others are indicated in red and blue, respectively. Reference sequences are shown in black.

An additional 691 partial sequences from the Philippines were available from the HIV sequence database. We next sought to investigate how the newly characterized sequences related to these partial sequences. Two phylogenetic trees were constructed for the partial *pol* and *env* sequences (Fig. 4). Similar to the previous analysis, all partial *pol* and *env* sequences of the new CRF01_AE viruses still formed a tight cluster together with sequences previously reported from the Philippines (Fig. 4a), or *per se* (Fig. 4b). Three new subtype B sequences were intermingled with subtype B reference sequences and were as divergent as previously reported partial *pol* and *env* sequences (Fig. 4). Taken together, new CRF01_AE sequences from this study appear to form a closely related cluster among CRF01_AE sequences, together with, or without, previously reported CRF01_AE sequences, suggesting that they share the same most recent common ancestor (MRCA), while other previously reported CRF01_AE sequences formed a distinct cluster, suggesting that they were decendents from another MRCA. New subtype B sequences were intermingled with other subtype B sequences, indicating that they were the results of multiple introductions into the Philippines.

### Ongoing extensive recombinants between CRF01_AE and subtype B

Detailed recombination analysis of six recombinant NFLG sequences showed that five CRF01/B recombinants had distinct recombination patterns between CRF01_AE and subtype B (Fig. 2). Among 18 recombination breakpoints, only two sites were shared among three viruses (1001, 1005 and 1011), suggesting that these recombinants were newly generated and had not yet spread as widely as circulating strains in the population.

**Fig. 5.**
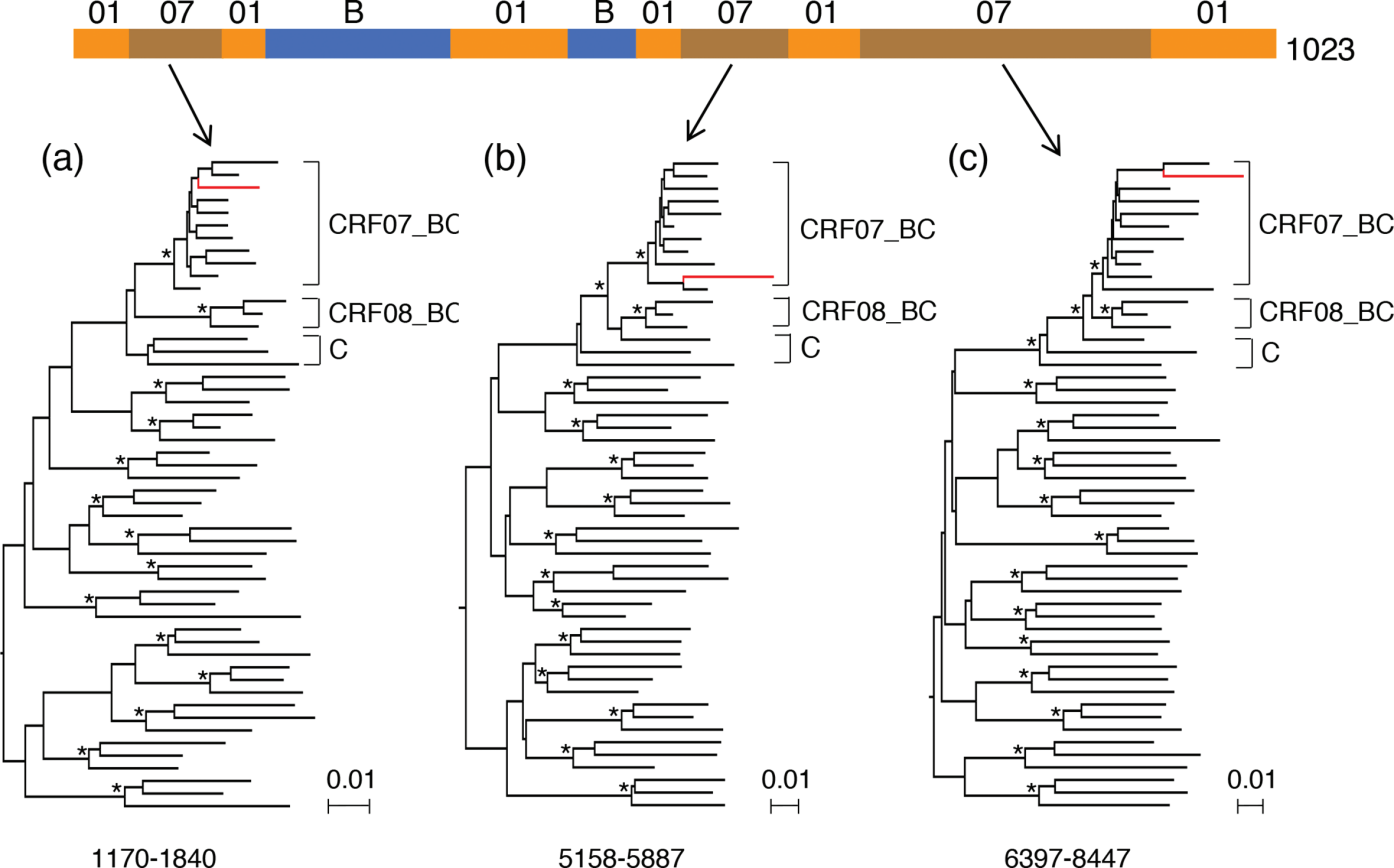
Identification of CRF07_BC-like sequences in the 1023 genome. Three subtype C-origin fragment sequences (a, b and c) in the 1023 genome were aligned together with seven additional CRF07_BC reference sequences. Phylogenetic trees were constructed using the neighbuor-joining method and the Kimura two-parameter model. The position of each recombinant region based on itse location in the HXB2 genome is indicated at the bottom of the tree. The scale bar represents 0.01 nucleotide substitutions per site. The sequences from 1023 are shown in red.

The NFLG sequence from 1023 was a complicated recombinant among CRF01_AE, C and subtype B in the initial analysis, with six fragments from CRF01_AE, three from subtype C and two from subtype B (Fig. 2). While including the CRF07_BC and CRF08_BC reference sequences, which are exclusively predominant in China [25], all three subtype C region sequences clustered tightly only with CRF07_BC sequences and not with CRF08_BC sequences or pure subtype C sequences (Fig. 5). This indicates that all three region sequences were specifically derived from CRF07_BC viruses rather than from other subtype C viruses. Taken together, these results show that a high percentage (26 %) of NFLG sequences were recombinants, and extensive recombination had been ongoing between CRF01_AE and other genotype viruses.

### Timing of the introduction of CRF01_AE in the Philippines

**Fig. 6.**
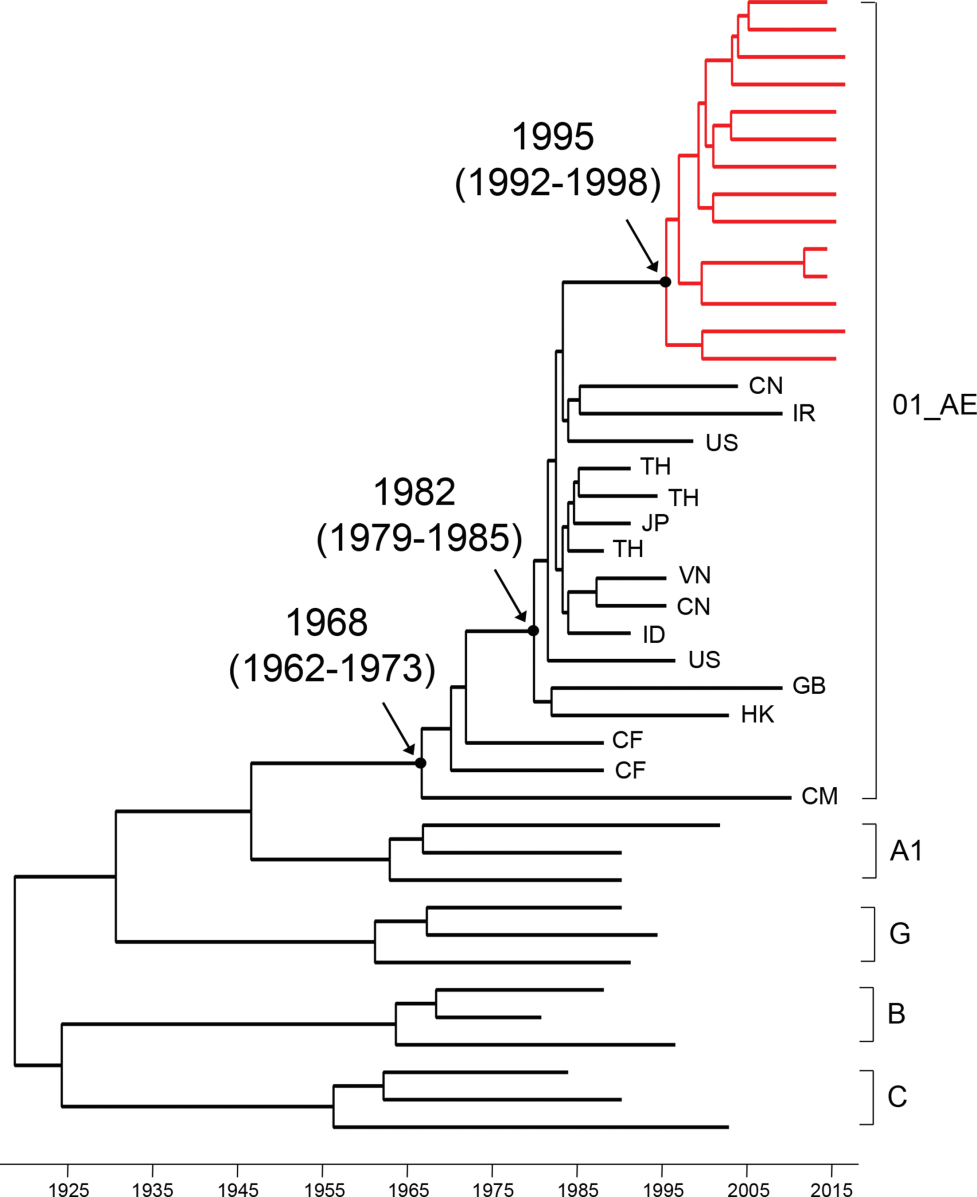
Estimated time of introduction of CRF01_AE in the Philippines. Maximum-clade credibility trees were generated for the subtype CRF01_AE NFLG sequences (red) using the Bayesian MCMC approach implemented in beast1.8.2. Each Markov chain Monte Carlo (MCMC) analysis was run for 50 million steps and sampled every 10 000 states. Posterior probabilities were calculated with a 10 % burn-in and checked for convergence using Tracer v1.6. FigTree 1.4.2 was used for visualization of the annotated trees. The mean time and 95 %HPD of the most common ancestor (tMRCA: year) are shown for the key notes based on relaxed (non-correlated log-normal) molecular clocks under HKY nucleotide substitution models in a gamma-distribution of among-site rate heterogeneity with four rate categories (HKY+γ4). All posterior probability values for key nodes are 1.0.

To estimate the timing of the introduction of CRF01_AE viruses in the Philippines, we generated a maximum clade credibility (MCC) tree with NFLG sequences of 14 CRF01_AE sequences from this study, 16 CRF01_AE reference sequences from different countries and 12 group M reference sequences (A1, B, C and G) using beast v1.8.2 as previously described [20, 26]. All CRF01_AE sequences from the Philippines formed unique independent subclusters within the CRF01_AE sequence clade (Fig. 6). The time to the most recent common ancestor (tMRCA) for CRF01_AE was estimated using the relaxed molecular clock with HKY substitution. Phylogenetic reconstruction using this model showed that tMRCA for CRF01 viruses was 1995 [95 % highest posterior density (HPD): 1992–1998], which is about 13 years later than CRF01_AE was introduced into Asia (1982, 1979–1985) and 27 years later than CRF01_AE was introduced into Africa (1968, 1962–1973). This result indicates that CRF01_AE was introduced into the Philippines quite recently and evolved into a unique subpopulation after its introduction in the mid-1990s.

### Prevalence of drug-resistant viruses in the Philippines

Analysis of NFLG sequences also allowed us to estimate the frequency of drug-resistant viruses in the Philippines. After the raw reads were analysed using HyDRA [21], we detected four major drug-resistant mutations (DRMs) in four patients (1005, 1008, 1022 and 1023) (Table 2). The prevalence of drug-resistant viruses (17 %, 4 of 23 samples) in these samples from the Philippines is relatively high compared to that in other countries [27, 28]. The highest percentage of the DRM in the viral population was 12.7 % for M184I in 1005. The percentages of the three other DRMs were very low – from 2.2 to 2.6 %. All those DRMs were present in <20 % of the viral population and were probably undetectable by the conventional Sanger population sequencing method [29, 30]. Multiple DRMs were not detected in any participants, and no DRMs to integrase inhibitors were detected in any participant.

**Table 2.** Detection of drug resistance mutations among viruses in the Philippines

Virus	Subtype	Percentage of each mutation in the viral population			
		PI	NRTI		NNRTI
		M46I	M184I	L210W	G190E
1005	URF_01B		12.7		
1008	CRF01_AE				2.2
1022	B			2.6	
1023	URF_0107B	2.6			

PI, protease inhibitor; NRTI, nucleoside RT inhibitor; NNRTI, non-nucleoside RT inhibitor.

## Discussion

Analysis of NFLG sequences from 23 HIV-1-infected individuals in the Philippines showed that CRF01_AE was the most predominant (61 %), while subtype B accounted for only 13 % of the virus population. We also found a high percentage (26 %) of recombinants. This is significantly different from what was previously reported [5–8], which showed 70 % subtype B, 20 % CRF01_AE and only small percentages of other recombinants. Although our study sample size was relatively small, the high percentage of CRF01_AE and low percentage of subtype B indicate a sharp change in the genotype distribution in the Philippines. Our results are in agreement with a more recent study which showed CRF01_AE at 77 % and subtype B at 22 % by analysing only a partial *pol* gene sequence [9], suggesting a dramatic shift is indeed happening in the Philippines. It will be interesting to determine what has caused such a dramatic shift in viral genotype in the Philippines, by conducting expanded phylogenetic studies of recently transmitted HIV from individuals with characterized demographic and risk factor information. The high percentage of CRF01_AE among the samples collected mainly from the MSM population in different provinces and cities in the Philippines suggests that CRF01_AE has spread widely through sexual transmission among MSMs. The significantly higher percentage of recombinants in this study than those previously reported [4, 8, 9] demonstrates that the proportion of recombinants in a region can be significantly underestimated using only partial genome sequences [31–34]. An accurate distribution of HIV-1 subtypes, CRFs and unique recombinants can only be reliably estimated by NFLG sequences.

All new CRF01/B recombinants had unique recombination patterns, and only a few recombinant breakpoints were shared among the six recombinants. This indicates that a high level of recombination is ongoing between these two predominant co-circulating genotypes (CRF01_AE and subtype B) in the Philippines. Interestingly, one virus (1023) from an MSM participant was a recombinant among CRF01_AE, CRF07_BC and subtype B. CRF07_BC originated from southwestern China [35] and has quickly become one of the main circulating CRFs and subtypes in that country [36]. CRF07_BC had not previously been identified in the Philippines. The identification of CRF07_BC-like sequences in three regions in the viral genome of 1023 strongly suggests that CRF07_BC was introduced into the country but is present at a level too low to be detected.

The tight cluster of CRF01_AE NFLG sequences suggested that these were the result of a single introduction and evolved into a unique viral population in the Philippines after its introduction. Molecular evolution clock analysis of NFLG sequences showed that these CRF01_AE viruses were probably introduced into the Philippines around 1995. CRF01_AE originated in Central Africa [37], but was found to be most prevalent in Asian countries [38–40]. CRF01_AE was introduced into the Philippines about 10–15 years later than it was into other Asian countries including Thailand (late 1970s) [41], China (mid-1980s) [26] and Vietnam (early 1980s) [40]. One of the reasons may be because that the Philippines is geographically isolated from other neighbouring countries, leading to a relatively later introduction. Interestingly, one CRF01_AE virus from Japan falls into the same tight cluster as those from the Philippines (Fig. 3). However, it is unclear by which routes that these viruses were transmitted from between countries due to the lack of epidemiological information.

Four major DRMs were found in four individuals, accounting for 17 % (4/23) of 23 treatment-naïve HIV-1-infected individuals in this study. This prevalence is much higher than those in other Asian countries such as China (3.8 %) [27] and Thailand (2.0 %) [28], but is at the high end of the prevalence scale (13.5 –20 %) in Western Europe and North America [42, 43]. One of the probable reasons for such a high prevalence in the treatment-naïve population of the Philippines is that the lower-frequency DRMs in the samples were easily detected by NGS. Three of four DRMs were present at ~2 %, while the fourth was present only at 12.7 %. Compared to conventional Sanger sequencing, which generally detects only those DRMs present at >20 % in the viral population [44–46], NGS can detect as low a level as ~1 % of DRMs [47]. This further confirms the importance of detection or monitoring DRMs using more sensitive methods.

The results from this study underscore the importance of NFLG sequence analysis in determination of the distribution of HIV-1 genotypes across diverse geographic regions for accurate detection of recombination patterns in recombinant HIV-1 genomes. The extensive recombination and marked increase of recombinants in a population will significantly increase the complexity of genetic variation [48], which may have important implications in vaccine development and patient treatment. Understanding dramatic shifts among HIV-1 subtypes, CRFs and unique recombinants, as well as the prevalence of drug-resistant viruses in a population, will be important for better epidemic control, development of effective vaccines and better treatment of HIV-1-infected individuals
